# Association of elevated circulating GDF15 and risk in acute retinal artery occlusion

**DOI:** 10.3389/fneur.2026.1707237

**Published:** 2026-03-16

**Authors:** Fangyuan Zhu, Hang Liu, Ruobing Shi, Kaichao Xia, Yuedan Wang, Liang Hu, Ting Chen, Ying Li, Anhuai Yang, Xuan Xiao

**Affiliations:** 1 Department of Ophthalmology, Renmin Hospital of Wuhan University, Wuhan, China; 2 Department of Clinical Laboratory, Institute of Translational Medicine, Renmin Hospital of Wuhan University, Wuhan, China

**Keywords:** cross-sectional study, growth differentiation factor 15, retinal artery occlusion, risk factor, serum biomarkers

## Abstract

**Purpose:**

We aimed to investigate the association between circulating growth differentiation factor-15 (GDF15) levels and retinal artery occlusion (RAO), and to assess the diagnostic performance of GDF15 for discriminating RAO patients from controls.

**Materials and methods:**

In this cross-sectional study, we quantified serum GDF15 levels using enzyme-linked immunosorbent assay (ELISA). After performing propensity score matching with age and sex adjustment, we conducted univariate and multivariate analyses to describe RAO risk factors. Subsequently, multivariable logistic regression combined with restricted cubic spline analysis was performed to assess the significance of GDF15 in RAO risk evaluation.

**Results:**

The results showed that GDF15 levels in patient was significantly increased in serum (median: 587.89 pg./mL vs. 331.54 pg./mL, *p* < 0.001) and aqueous humor (median: 442.8 pg./mL vs. 81.21 pg./mL, *p* < 0.01) of patients. Univariate and multivariate analyses identified triglyceride (TG), glucose (Glu), and GDF15 as independent risk factors for RAO. Multivariable linear regression analysis revealed that TG and Glu were positively correlated with GDF15 levels, whereas estimated glomerular filtration rate (eGFR) and triglyceride–Glu (TyG) were inversely correlated. The multiparameter combination (GDF15, TyG index, Glu, TG, neutrophil) demonstrated superior diagnostic performance for RAO (area under the curve, AUC = 0.92) compared with individual biomarkers, each of which showed moderate discriminative ability.

**Conclusion:**

Our findings indicate that elevated GDF15 levels are significantly associated with the incidence of RAO. GDF15 exhibited acceptable diagnostic accuracy as a single marker, and the inclusion of GDF15 in a multiparameter diagnostic model significantly improved discrimination, highlighting its potential as a biomarker for RAO.

## 1 Introduction

Retinal artery occlusion (RAO) is represented as a critical ophthalmic emergency characterized by sudden, painless unilateral vision loss, with an incidence of 1–2 cases per 100,000 individuals (1). Typically, RAO is caused by micro-embolic obstruction of the central or branch retinal artery occlusion (CRAO or BRAO) (2), progressing rapidly from inner retinal edema to irreversible retinal atrophy (3). This cascade often results in irreversible visual impairment within 4.5 h (4). RAO shares vascular risk factors with ischemic stroke, including hypertension, diabetes, and cardiovascular diseases, reflecting similar pathophysiological mechanisms (5–7). Despite its severe prognosis, RAO lacks methods for early risk identification and effective treatments. Novel interventions, such as intra-arterial thrombolysis, are limited by narrow therapeutic windows and inconsistent outcomes (8). The multifaceted interaction of oxidative stress, inflammation, and vascular dysfunction underscores the critical necessity of reliable biomarkers to enable early diagnosis and intervention, potentially mitigating retinal ganglion cells (RGCs) loss and improving clinical management of this vision-threatening condition.

Growth differentiation factor 15 (GDF15), a stress-responsive cytokine member belonging to the transforming growth factor-beta (TGF-*β*) superfamily, is upregulated in response to inflammation, hypoxia, and oxidative stress (9). The elevation of GDF15 levels has been extensively investigated and shown to correlate with ischemic stroke incidence and patient prognosis (10, 11). In ophthalmic diseases, GDF15 has been proved as a protective factor in RGCs apoptosis progression (12), a condition sharing neurodegenerative features with RAO. Considering its dual role in vascular and neurodegenerative pathways, GDF15 has become a compelling candidate for recent RAO research. Investigating GDF15 contributes to understanding RAO pathophysiology and informs novel diagnostic and therapeutic strategies.

Given the promising diagnostic utility of GDF15 in ischemic and ocular diseases, we hypothesize that GDF15 plays a critical role in RAO pathogenesis. This study systematically evaluates the relationship between serum GDF15 levels with combined indicators and the onset of RAO through systematic biomarker analysis. Our preliminary data revealed a positive correlation between elevated GDF15 concentrations and RAO incidence, suggesting the potential of GDF15 as a diagnostic biomarker. These elevated levels likely reflect underlying retinal ischemic processes, thereby enabling rapid clinical identification. Combination of GDF15 and other biomarkers may enhance diagnostic precision, addressing the unmet need for reliable early detection tools in RAO.

## 2 Materials and methods

### 2.1 Data source and study population

**Figure 1:**
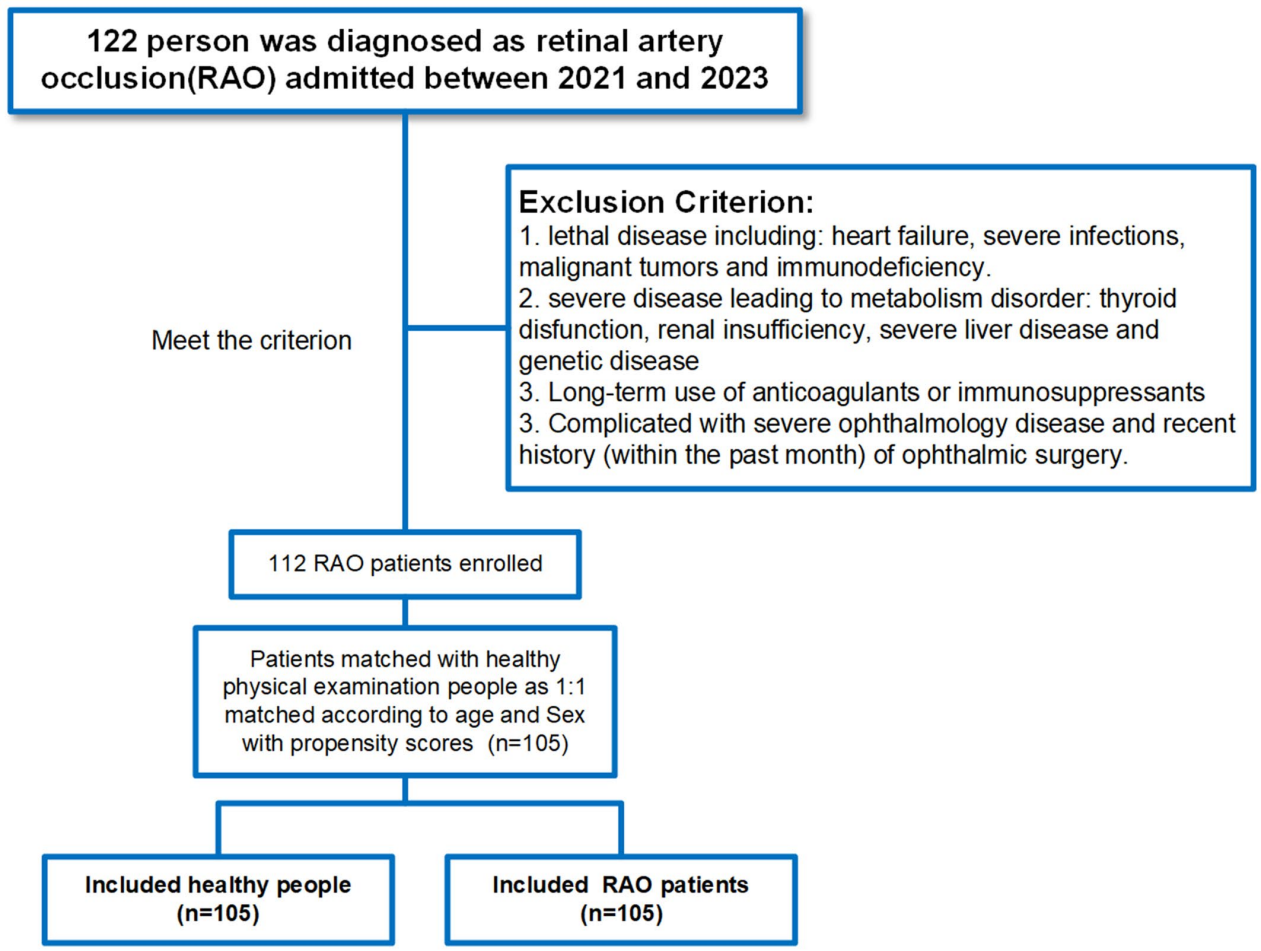
Flowchart of RAO patient selection.

This cross-sectional study enrolled 122 patients who were newly diagnosed with RAO at Renmin Hospital of Wuhan University between June 2021 and October 2023. All patients underwent a standardized ophthalmologic evaluation, including assessments of visual acuity, slit-lamp biomicroscopy, fundus examination, and optical coherence tomography. For patients with suspected RAO, fluorescein fundus angiography was performed to confirm the diagnosis and determine the location and severity of retinal arterial occlusion. For control comparisons, 105 healthy individuals matched for age and sex distribution were prospectively selected during the identical recruitment period. Subgroup analyses were conducted in the PSM cohort by stratifying participants according to hypertension and diabetes status. Circulating GDF15 levels were compared between RAO patients and controls within each subgroup (Supplementary Table 1). These participants had no history of ocular or systemic vascular diseases and underwent routine ophthalmologic examination to confirm normal retinal status. The detailed inclusion and exclusion criteria for both RAO patients and healthy controls are presented in Figure 1.

### 2.2 Study definition and clinical assessment

The RAO diagnosis was established according to standardized clinical criteria, including: (1) acute-onset painless monocular vision loss, (2) demonstrable relative afferent pupillary defect, (3) characteristic fundoscopic findings of retinal ischemia (typically manifesting as edema with foveal cherry-red spot), and (4) angiographic confirmation of delayed arterial perfusion on fluorescein fundus angiography (13). Hypertension was defined as: (1) recent use of antihypertensive pharmacotherapy for blood pressure control or (2) persistent elevation of blood pressure (≥140/90 mmHg) documented on ≥2 distinct clinical measurements (14). Diabetes mellitus was defined as either: (1) physician-diagnosed diabetes with ongoing management or (2) meeting laboratory criteria according to present guidelines [fasting plasma glucose (Glu) ≥ 126 mg/dL and/or 2-h postprandial Glu ≥ 200 mg/dL] (15).

### 2.3 Sample collection and laboratory measurement

All participants were instructed to fast overnight before venous blood collection. Blood samples were drawn into EDTA-K_2_ containing tubes and analyzed for complete blood count parameters using an automated hematology analyzer (XN-9000; Sysmex, Japan). For biochemical assessments, blood samples were transferred to serum isolation tubes and subjected to natural clotting at 25 °C. The resulting serum was then analyzed by routine biochemical tests, including liver and renal function, lipid profile, and fasting blood glucose using an automated chemistry analyzer (ADVIA 2400; Siemens, Germany). The samples were then centrifuged at 3000 rpm for 15 min, the separated serum was aliquoted into 1.5 mL microcentrifuge tubes and stored at −80 °C until analysis. Aqueous humor samples were collected through limbal paracentesis using a 30-gage needle, aspirated 0.05–0.1 mL from the anterior chamber, and were cryopreserved at −80 °C for analysis. Control samples consisted of aqueous humor from cataract surgery patients. GDF15 concentrations in both serum and aqueous humor were quantified in batches by human enzyme-linked immunosorbent assay (ELISA) kit (DGD150; R&D Systems, United States).

### 2.4 RAO mouse model establishment and sample collection

Male C57BL/6 mice (8 weeks old, body weight 20–23 g) were housed with free access to food and water and maintained under controlled temperature and humidity conditions. All experimental protocols were conducted in accordance with the ethical guidelines outlined by the Association for Research in the United States and approved by the Animal Ethics Committee of Renmin Hospital of Wuhan University (Approval No. WDRM-20220305A). Mice were anesthetized using an isoflurane inhalation (induction of 3–4% and maintenance of 1.5–2%). Adequate anesthesia was confirmed by the absence of pedal withdrawal reflex. The unilateral pterygopalatine ophthalmic artery occlusion (UOPAO) mouse model was established as previously described (16). On day 7 after model establishment, mice were deeply anesthetized with sodium pentobarbital (150 mg/kg, i.p.) and euthanized by cervical dislocation as a secondary physical method to ensure death. Death was confirmed by cessation of respiration and heartbeat.

Blood samples were collected immediately through enucleation-induced orbital sinus bleeding. Following centrifugation, serum fractions were aliquoted and cryopreserved at −80 °C pending downstream analyses. Serum levels of growth GDF15 were measured using a commercial mouse-specific ELISA kit (E-EL-M0604; Elabscience, China) in accordance with the manufacturer’s instructions.

### 2.5 Statistical analysis

For continuous variables, data are presented as mean ± standard deviation (SD) for normally distributed variables and as median (interquartile range, IQR) for non-normally distributed variables, according to their distributional characteristics. Differences between the two groups were estimated either by the independent samples *t*-test or the Mann–Whitney *U* test, based on data distribution. Categorical variables were presented as frequencies and percentages. Univariate and multivariate logistic regression analyses were performed to identify potential risk factors. Candidate variables for the multivariable model were initially screened using univariate logistic regression (*p* < 0.05). Subsequently, among these candidates, collinearity was assessed using the variance inflation factor (VIF), and variables with a VIF > 5 were excluded to minimize multicollinearity. A restricted cubic spline model was employed to investigate the potential nonlinear association between GDF15 levels and RAO risk. Receiver operating characteristic (ROC) curve analysis was performed to evaluate the discriminative ability of GDF15 and other clinical blood indices, with AUC calculated to quantify diagnostic performance. All statistical analyses were performed using IBM SPSS Statistics version 26.0 (IBM Corp., Armonk, NY, United States), and graphical outputs were generated using GraphPad Prism version 8.0 (GraphPad Software, San Diego, CA, United States).

## 3 Results

### 3.1 Elevated GDF15 levels and distinct clinical characteristics in RAO patients

**Figure 2:**
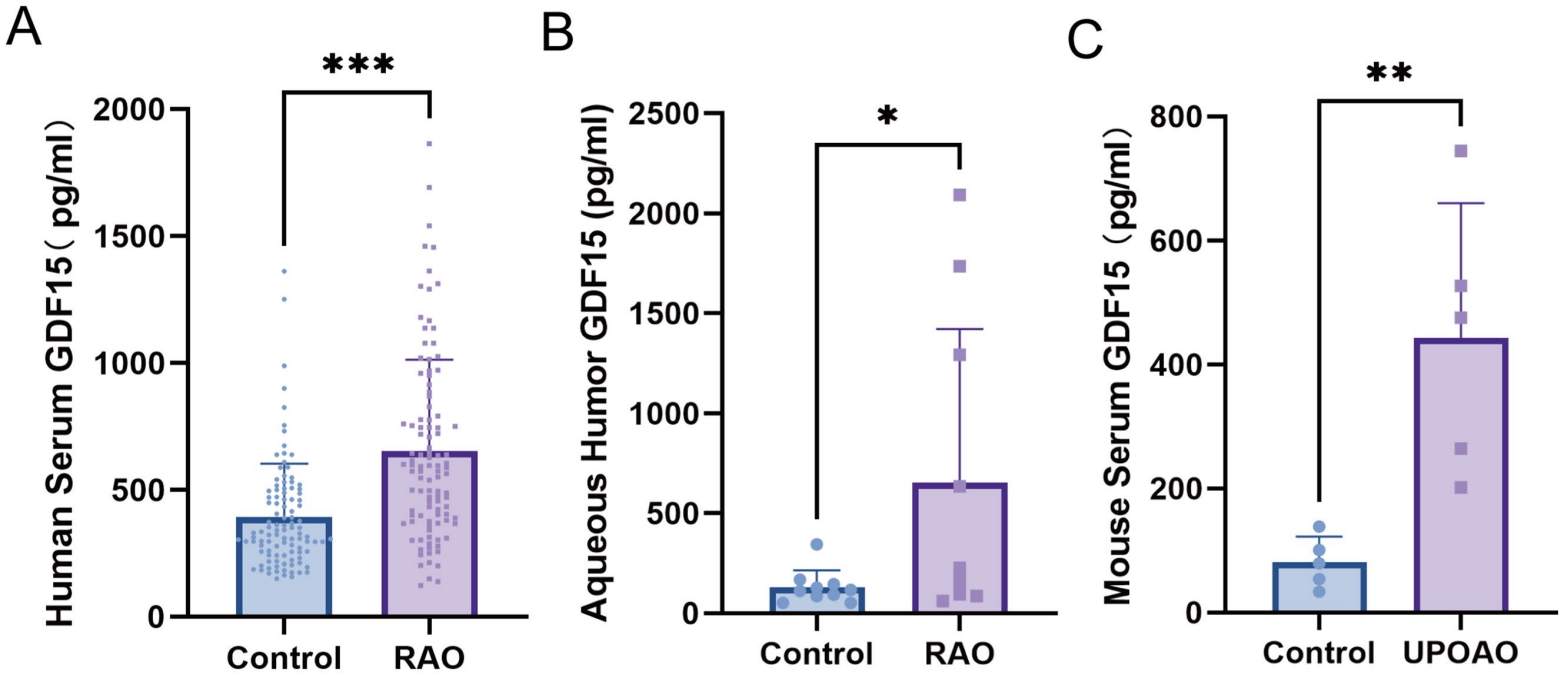
GDF15 level in **(A)** human serum, **(B)** human aqueous humor, **(C)** mouse serum. Abbreviation: GDF15: growth differentiation factor 15. UPOAO: unilateral pterygopalatine ophthalmic artery occlusion mouse model. *p < 0.05; **p < 0.01; ***p < 0.001.

Serum GDF15 levels were elevated in the RAO patients compared with healthy controls (median: 587.89 pg./mL vs. 331.54 pg./mL, *p* < 0.001), indicating a potential association between elevated circulating GDF15 levels and RAO occurrence (Table 1; Figure 2A). Analysis of aqueous humor samples from a subset of RAO patients and cataract patients revealed markedly higher intraocular GDF15 levels in RAO patients than those in controls, approximately fivefold higher (653.9 pg./mL vs. 129.8 pg./mL; Figure 2B). These findings were further validated *in vivo*, as serum GDF15 levels in a unilateral RAO mouse model were significantly elevated compared with sham-operated controls (442.8 pg./mL vs. 81.21 pg./mL; Figure 2C), supporting the clinical association between GDF15 and RAO. Baseline clinical characteristics of patients with CRAO and BRAO are summarized in Supplementary Table 2. Results showed that the GDF15 levels were 589.90 (378.67–775.62) pg./mL in the CRAO subgroup and 642.82 (425.99–1168.58) pg./mL in the BRAO subgroup.

**Table 1:** Baseline characteristics of the control group and the RAO group.

Characteristics	Control group *n* = 105	RAO group *n* = 105	*p* value
Clinical variables			
Male sex (%)	67.60	71.40	0.552
Age (years)	58 (54, 65)	59 (51, 67)	0.732
Hypertension (%)	20.40	58.10	<0.001
Diabetes (%)	4.80	14.30	0.021
Laboratory variables			
WBC (×10^9^/L)	5.66 (4.84, 7.02)	6.38 (5.30, 7.85)	0.019
Neu (×10^9^/L)	2.96 (2.42, 3.78)	3.67 (2.97, 4.76)	<0.001
Lym (×10^9^/L)	2.05 (1.74, 2.44)	1.82 (1.46, 2.34)	0.009
Mono (×10^9^/L)	0.44 (0.35, 0.54)	0.50 (0.39, 0.59)	0.015
ALT (U/L)	18.00 (14.00, 25.00)	18 (13.00, 27.00)	0.891
AST (U/L)	22.00 (19.00, 24.00)	18.50 (16.00, 24.00)	0.001
ALT/AST	0.87 (0.71, 1.09)	1.00 (0.76, 1.25)	0.018
Tch (mmol/L)	4.52 (4.12, 4.83)	4.50 (3.96, 5.23)	0.193
TG (mmol/L)	1.08 (0.89, 1.31)	1.57 (1.04, 2.05)	<0.001
HDL-ch (mmol/L)	1.33 (1.12, 1.48)	0.99 (0.85, 1.14)	<0.001
LDL-ch (mmol/L)	2.56 (2.27, 2.91)	2.81 (2.24, 3.34)	0.009
Tch/HDL-ch	3.39 (2.97, 3.88)	4.54 (3.76, 5.33)	<0.001
Urea (mmol/L)	5.16 (4.48, 6.13)	5.57 (4.79, 6.52)	0.024
Cr (μmol/L)	66.5 (58, 73.75)	71.00 (58, 80.75)	0.047
Urea/Cr	12.86 (11, 15.26)	11.84 (9.66, 14.88)	0.052
Glu (mmol/L)	4.85 (4.5, 5.15)	5.30 (4.90, 5.97)	<0.001
eGFR (mL/min/1.73 m^2^)	98.20 (93.33, 102.02)	95.57(86.32, 104.69)	0.034
TyG index	8.31 (8.243, 8.753)	8.868(8.361, 8.982)	<0.001
GDF15 (pg/mL)	331.54 (255.41, 492.49)	587.89(384.69, 809.78)	<0.001

Data are expressed as percent (n) or median (interquartile range). WBC, White blood cell; Neu, Neutrophil; Lym, Lymphocyte; Mono, Monocyte; ALT, Glutamic pyruvic transaminase; AST, Glutamic oxaloacetic transaminase; ALT/AST, Glutamic pyruvic transaminase to glutamic oxaloacetic transaminase ratio; Tch, Total cholesterol; TG, Triglycerides; HDL-ch, High-density lipoprotein cholesterol; LDL-ch, Low-density lipoprotein cholesterol; eGFR, Estimated glomerular filtration rate; Glu, Glucose; GDF15, Growth differentiation factor 15. TyG (Triglyceride–glucose index) is calculated using the formula: TyG index = ln[fasting triglycerides (mg/dL) × fasting glucose (mg/dL)/2].

Beyond GDF15, laboratory parameters revealed distinct inflammatory and metabolic alterations in the RAO cohort. White blood cell (WBC), neutrophil (Neu), and monocyte (Mono) counts were significantly elevated, while lymphocyte (Lym) counts were decreased, indicating evidence of systemic inflammation. Although alanine aminotransferase (ALT) levels were comparable between groups, RAO patients had lower aspartate aminotransferase (AST) levels (*p* = 0.001), leading to a higher ALT/AST ratio (*p* = 0.018). Atherogenic dyslipidemia was more pronounced in the RAO group, evidenced by higher levels of triglycerides (TGs, *p* < 0.001), low-density lipoprotein cholesterol (LDL-ch, *p* = 0.009), and total cholesterol to HDL-ch ratio (TCh/HDL-ch, *p* < 0.001), along with reduced high-density lipoprotein cholesterol (HDL-ch, *p* < 0.001). The triglyceride–glucose (TyG) index was significantly elevated in RAO patients (8.868 vs. 8.302, *p* < 0.001). Furthermore, renal function indices showed signs of vascular involvement: RAO patients had significantly higher fasting Glu (*p* < 0.001), urea (*p* = 0.024), and creatinine (Cr, *p* = 0.047) levels, accompanied by a lower estimated glomerular filtration rate (eGFR, *p* = 0.034), suggesting potential renal impairment or systemic vascular dysfunction (Table 1).

### 3.2 Association between serum GDF15 and demographic and clinical parameters

To investigate the association between serum GDF15 levels and RAO incidence, univariate logistic regression was initially performed. Collinearity diagnostics of variables included in the multivariable model are presented in Supplementary Table 3. In the multivariate logistic regression analysis, TG, Glu, and GDF15 levels remained independently associated with the risk of RAO after adjustment for potential confounders. Specifically, higher TG levels were significantly associated with an increased risk of RAO (OR = 11.988, 95% CI: 3.726–38.568, *p* < 0.001). Elevated Glu was also independently associated with RAO risk (OR = 3.663, 95% CI: 1.699–7.897, *p* = 0.001). In addition, GDF15 levels showed a significant and independent association with RAO (OR = 1.004, 95% CI: 1.002–1.005, *p* < 0.001). Hypertension, blood cell counts, urea, and eGFR were not independently associated with RAO after adjustment (*p* > 0.05; Table 2).

**Table 2:** Univariate and multivariate analyses for the risk factors of RAO.

Characteristics	OR (95%CI)	*p* value	OR (95%CI)	*p* value
Male, sex (%)	0.835(0.464, 1.505)	0.552		
Age (years)	0.984(0.956, 1.012)	0.269		
Hypertension (%)	5.413(2.922, 10.028)	<0.001	0.479(0.205, 1.119)	0.089
Diabetes (%)	3.300(1.153, 9.445)	0.031	2.708(0.506, 14.489)	0.244
WBC (×10^9^/L)	1.265(1.056, 1.517)	0.011	1.164(0.912, 1.486)	0.222
Neu (×10^9^/L)	1.700(1.3, 2.223)	<0.001		
Lym (×10^9^/L)	0.580(0.363, 0.929)	0.023		
Mono (×10^9^/L)	8.352(1.417, 49.244)	0.018		
ALT (U/L)	1.019(1, 1.038)	0.052		
AST (U/L)	1.006(0.979, 1.033)	0.678		
Tch (mmol/L)	1.368(0.928, 2.016)	0.109		
TG (mmol/L)	10.779(4.615, 25.176)	<0.001	11.988(3.726, 38.568)	<0.001
Urea (mmol/L)	1.316(1.062, 1.631)	0.012	0.912(0.653, 1.275)	0.591
Glu (mmol/L)	4.112(2.326, 7.269)	<0.001	3.663(1.699, 7.897)	0.001
eGFR (mL/min/1.73 m^2^)	0.969(0.944, 0.994)	0.021	0.993(0.967, 1.020)	0.600
TyG index	23.032(8.766, 60.520)	<0.001		
GDF15 (pg/mL)	1.004(1.002, 1.005)	<0.001	1.004(1.002, 1.005)	<0.001

WBC, White blood cell; Neu, Neutrophil; Lym, Lymphocyte; Mono, Monocyte; ALT, Glutamic pyruvic transaminase; AST, Glutamic oxaloacetic transaminase; Tch, Total cholesterol; TG, Triglycerides; Glu, Glucose; eGFR, Estimated glomerular filtration rate; GDF15, Growth differentiation factor 15. TyG (triglyceride–glucose index) is calculated using the formula: TyG index = ln[fasting triglycerides (mg/dL) × fasting glucose (mg/dL)/2]; Variables with *p* < 0.05 in univariate analysis and without multicollinearity were included in the multivariate model.

**Figure 3:**
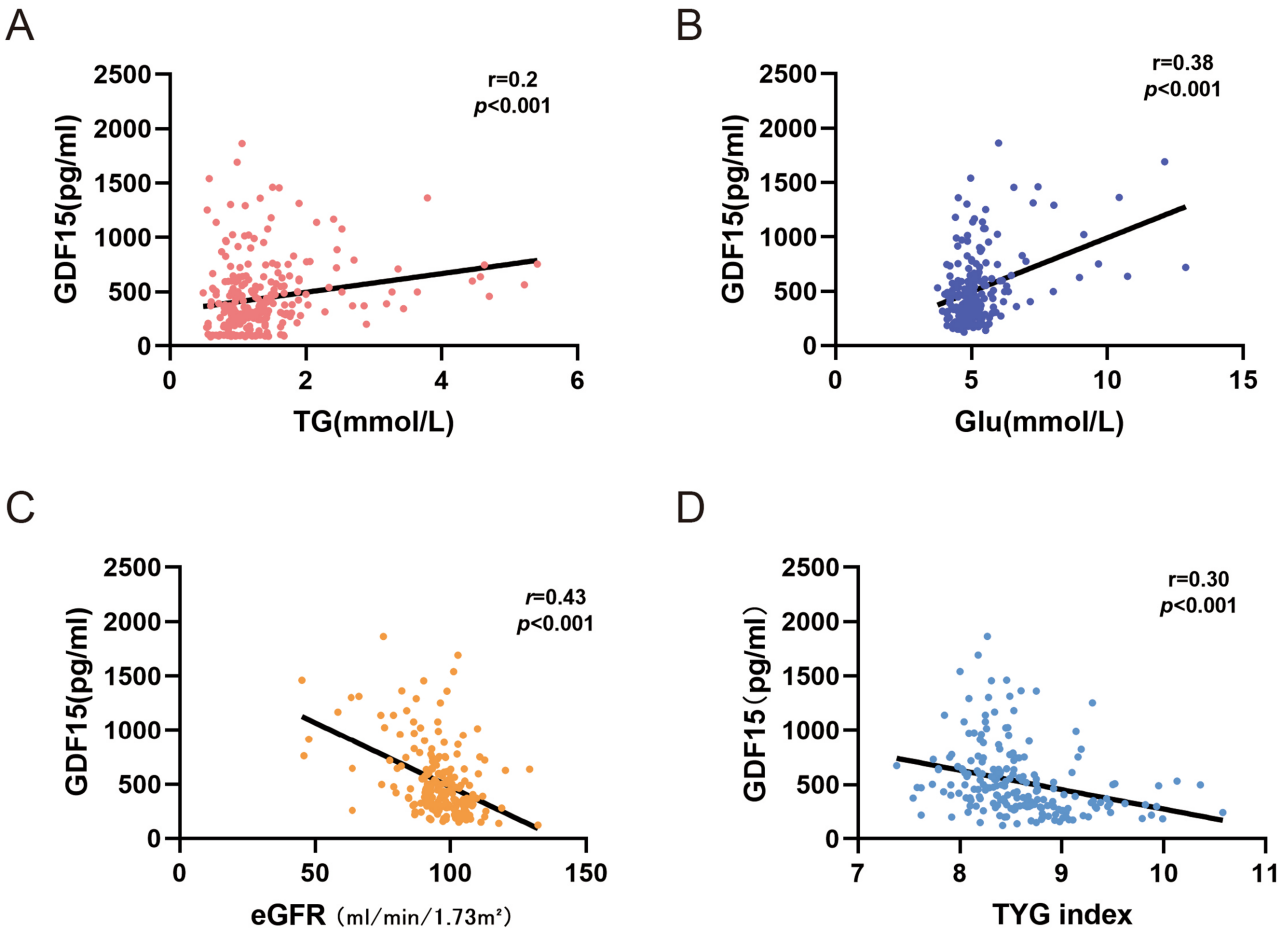
Correlates of GDF15 levels in Spearman linear regression analysis. **(A)** association between GDF15 and triglycerides (TG). **(B)** association between GDF15 and glucose (Glu). **(C)** association between GDF15 and estimated glomerular filtration rate (eGFR). **(D)** association between GDF15 and triglyceride–glucose (TYG) index. TG, Triglycerides Glu; Glucose eGFR, estimated Glomerular Filtration Rate TYG index; Triglyceride-Glucose Index.

Univariate Spearman linear regression analyses were conducted to further evaluate the relationship between GDF15 and clinical parameters. Serum GDF15 levels showed significant positive correlations with Glu (*R* = 0.38, *p* < 0.001) and TG (*R* = 0.20, *p* < 0.001; Figures 3A,B). In contrast, GDF15 was negatively associated with eGFR (*R* = −0.44, *p* < 0.001) and the TyG index (*R* = −0.29, *p* < 0.001; Figures 3C,D). These findings suggest a close link between elevated GDF15 and both dysregulated Glu–lipid metabolism and impaired renal function in RAO patients.

### 3.3 Logistic regression between serum GDF15 levels and RAO risk

To evaluate the relationship between circulating GDF15 levels and the RAO risk, all participants were stratified into quartiles based on serum GDF15 concentrations (Q1: ≤ 297.46 pg./mL; Q2: 297.47–442.64 pg./mL; Q3: 442.65–638.15 pg./mL; and Q4: > 638.15 pg./mL) as shown in Table 3. In the unadjusted (crude) model, individuals in the highest GDF15 quartile (Q4) demonstrated a markedly increased risk of RAO compared with those in the lowest quartile (Q1), with an OR of 12.60 (95% CI: 4.59–32.02). This association remained robust and further intensified following adjustment for potential confounding variables. In Model 1 (adjusted for age and sex), the OR for Q4 increased to 22.88 (95% CI: 7.01–74.67). In the fully adjusted Model 2 (additionally adjusted for age, sex, WBC, Neu, Lym, Mono, AST, ALT/AST, TG, HDL-ch, LDL-ch, Tch/HDL-ch, urea, Cr, eGFR, and Glu), the OR reached 65.69 (95% CI: 6.71–643.26), indicating a strong and independent association between elevated serum GDF15 levels and RAO onset.

**Table 3:** Logistic regression analysis of serum GDF15 levels for RAO patients.

GDF15 quartile	*N*	GDF15 (pg/mL)	OR (95%CI)		
			Crude	Model 1	Model 2
Quartile 1	52	≤297.46	Reference	Reference	Reference
Quartile 2	53	297.46–442.64	2.129(0.926, 4.893)	2.368 (0.914, 6.134)	4.354(0.788, 24.096)
Quartile 3	53	442.64–638.15	3.361(1.469, 7.687)	5.034(1.817, 13.951)	21.629(3.696, 126.527)
Quartile 4	52	≥638.15	12.601(4.59, 32.016)	22.876(7.008, 74.674)	65.692(6.709, 643.264)
*β*			2.534	3.13	4.185
SE			0.476	0.604	1.164
*P* for trend			<0.001	<0.001	<0.001

Crude: no adjustment. Model 1: Adjusted for sex, age, hypertension, and diabetes. Model 2: Adjusted for the same variables as Model 1, WBC, Neu, Lym, Mono, AST, ALT/AST, TG, HDL-ch, LDL-ch, Tch/HDL-ch, Urea, Cr, eGFR, and Glu.

### 3.4 Nonlinear association between serum GDF15 levels and RAO risk

**Figure 4:**
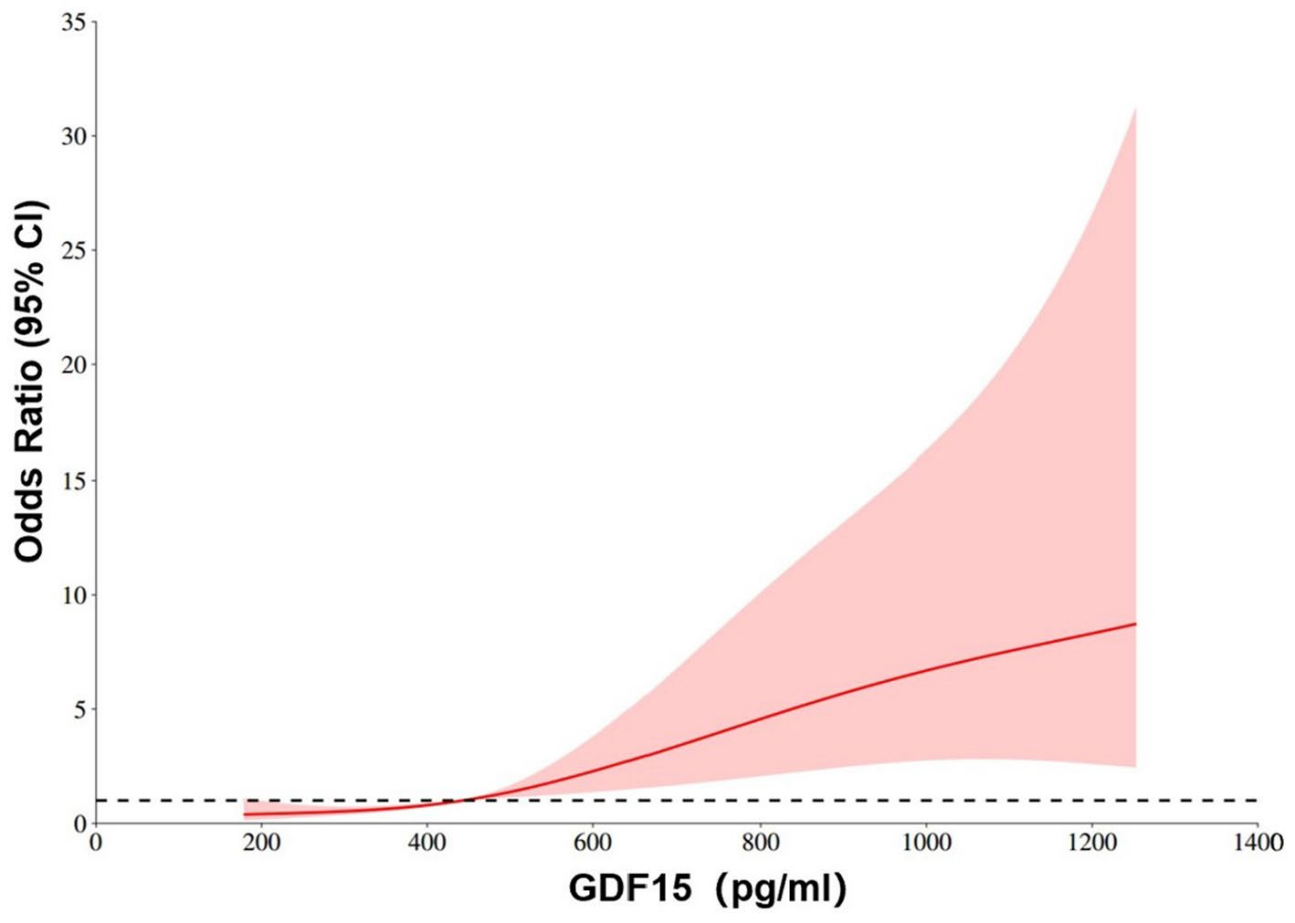
Restricted cubic spline model of the ORs of RAO patients with serum GDF15 levels. The dashed lines represent the 95% confidence intervals.

Figure 4 depicts the restricted cubic spline model assessing the nonlinear association between serum GDF15 levels and RAO risk. The red-shaded area represents the 95% confidence interval, while a threshold value of 442.642 pg./mL is indicated. The analysis demonstrated a positive, dose-dependent relationship between circulating GDF15 and RAO risk, supporting the hypothesis that elevated GDF15 levels may serve as an independent and nonlinear risk marker for RAO development. The overall trend of results was statistically significant, indicating that GDF15 was strongly associated with disease risk (*p* for overall <0.001), and GDF15 and OR showed a linear relationship (*p* for nonlinear = 0.275).

### 3.5 ROC curve analysis of GDF15 and other variables for RAO diagnosis

**Figure 5:**
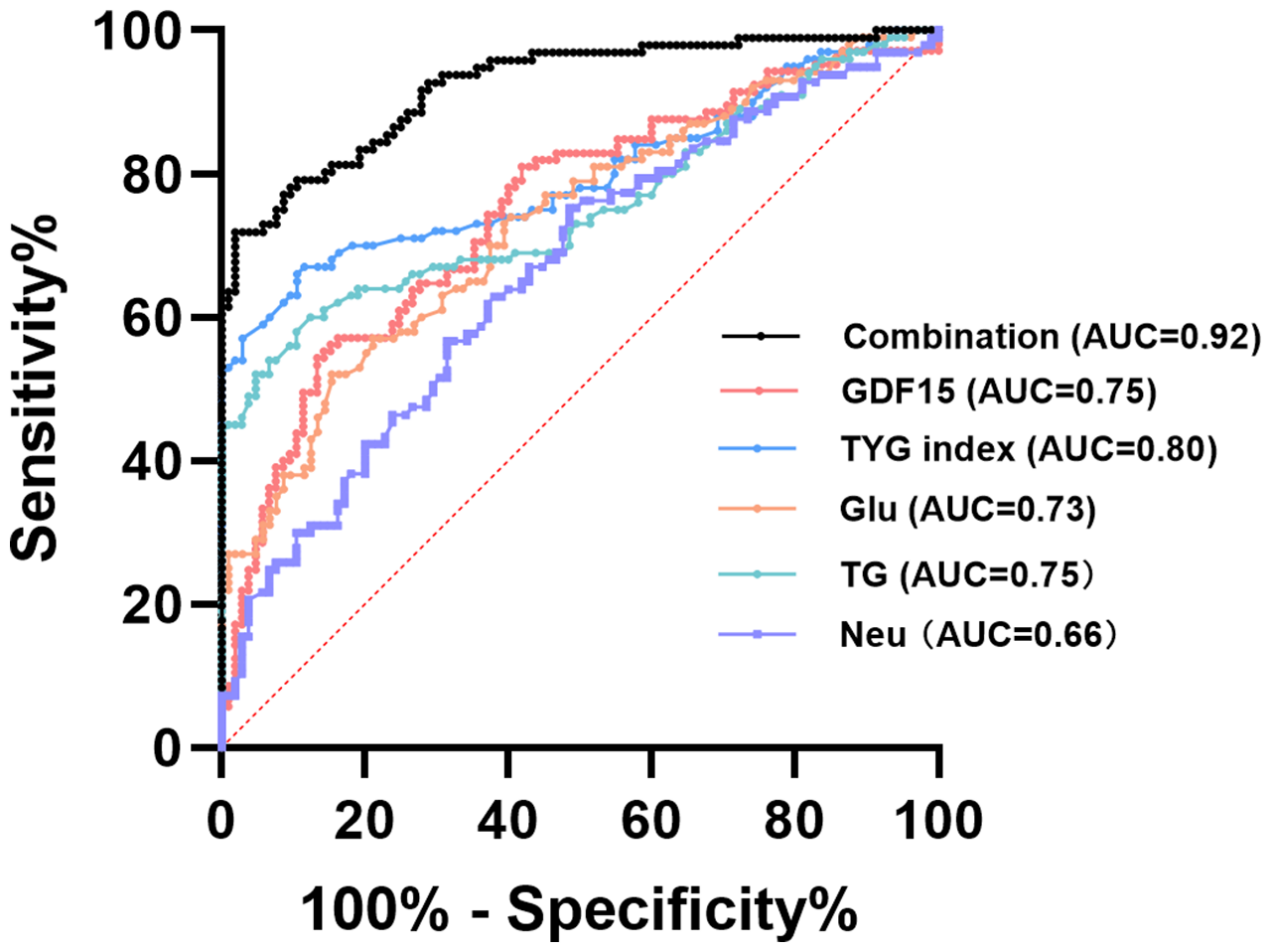
ROC curve analysis of GDF15 combined with laboratory indexes in the diagnosis of RAO. TG, Triglycerides Glu; Glucose eGFR, estimated Glomerular Filtration Rate; TYG index, triglyceride-glucose index; Neu, Neutrophil; Combination: GDF15+Glu+TG+Neu + TYG index.

To evaluate the diagnostic performance of serum GDF15 and other relevant clinical variables in identifying patients with RAO, ROC curve analysis was conducted (Table 4, Figure 5). The AUC for serum GDF15 was 0.748 (95% CI: 0.681–0.814), with an optimal cut-off value of 553.4 pg./mL, indicating moderate diagnostic utility. Among other variables, the AUCs were as follows: Glu, 0.730 (95% CI: 0.662–0.800); TG, 0.754 (95% CI: 0.686–0.823); HDL-ch, 0.818 (95% CI: 0.761–0.875); Neu, 0.662 (95% CI: 0.588–0.737); and TyG index, 0.798 (95% CI: 0.734–0.861). Notably, when these biomarkers were integrated into a combined predictive model, the diagnostic accuracy markedly improved, yielding an AUC of 0.922 (95% CI: 0.886–0.959). These results indicate that, while GDF15 alone demonstrates moderate predictive capability, the combination with metabolic and inflammatory markers substantially enhanced RAO diagnostic performance.

**Table 4:** ROC curve analysis of GDF15 levels combined with laboratory indexes in the diagnosis of RAO.

Variables	AUC	Sensitivity%	Specificity%	95%CI	Youden index	Cut-off
GDF15	0.748	54.29	86.67	0.681, 0.814	0.409	553.400 pg./mL
Glu	0.730	52.00	84.62	0.662, 0.800	0.366	5.285 mmol/L
TG	0.754	60.00	87.62	0.686, 0.823	0.476	1.425 mmol/L
HDL-ch	0.818	51.00	96.19	0.761, 0.875	0.472	0.995 mmol/L
Neu	0.662	75.26	51.43	0.588, 0.737	0.267	2.975 ×10^9^/L
TyG index	0.798	67.00	88.46	0.734, 0.861	0.555	8.628
Combination	0.922	71.88	98.08	0.886, 0.959	0.699	–

Glu, Glucose; TG, Triglycerides; HDL-ch, High-density lipoprotein cholesterol; Neu, Neutrophil; Combination, GDF15 + glucose + triglycerides + high-density lipoprotein cholesterol + neutrophil + TyG. TyG (triglyceride–glucose index) is calculated using the formula: TyG index = ln[fasting triglycerides (mg/dL) × fasting glucose (mg/dL)/2].

## 4 Discussion

RAO is a vision-threatening ischemic event that shares vascular risk factors and pathophysiological mechanisms with ischemic stroke, but lacks effective early diagnostic strategies(5). GDF15, a cytokine classified within the TGF-*β* superfamily, is notably elevated and triggered by cellular stressors such as inflammation, low oxygen levels, and oxidative damage (17). Previous researches have consistently demonstrated the risk and prognostic significance of GDF15 in ischemic stroke and cardiovascular diseases (18). In the present study, we observed a significant positive association between serum GDF15 levels and the prevalence of RAO. Notably, both the logistic regression and restricted cubic spline analyses revealed a dose-dependent relationship, suggesting that elevated GDF15 levels not only reflect the presence of RAO but also indicate disease impairment. These results conclusively validate the potential utility of GDF15 as a non-invasive biomarker for RAO.

Our study identified significantly elevated serum GDF15 levels in RAO patients, consistent with UK Biobank proteomic data showing increased GDF15, particularly in individuals with RAO and concurrent ischemic stroke (19). ROC analysis demonstrated that incorporating GDF15 into a panel of conventional biomarkers significantly improved diagnostic performance for RAO, as reflected by a higher AUC for the combined model compared with GDF15 alone. ROC analysis demonstrated that incorporating GDF15 into a panel of conventional biomarkers significantly improved diagnostic performance for RAO, as evidenced by a higher AUC for the combined model compared with GDF15 alone and with each conventional biomarker. Notably, RAO patients showed approximately five-fold higher GDF15 concentrations in aqueous humor compared with controls in this study, exceeding the magnitude of serum elevation. Under normal conditions, GDF15 expression is low in RGCs and aqueous humor but is markedly upregulated following RGC ischemia or injury, potentially accompanied by retinal barrier disruption and release into intraocular fluid (20). Elevated GDF15 levels have been observed in both systemic circulation and aqueous humor in glaucoma, which shares the same ischemia–reperfusion pathology of RGCs (21). RAO involves both vascular occlusion and ischemic death of RGCs, and previous studies have shown that both vascular smooth muscle cells and RGCs can release GDF15 in response to hypoxic stress (12, 22). Therefore, we hypothesize that the elevated aqueous humor GDF15 may originate not only from systemic circulation but also from local expression and secretion by RGCs. Supporting this, an unpublished data from our group demonstrate upregulated GDF15 expression in RGCs of RAO mice model after retinal ischemia–reperfusion impairment. Researches have established that GDF15 can directly inhibit RGCs apoptosis (12, 23). Elucidating the interaction between GDF15 and retinal ganglion cells during the course of RAO represents an important direction for future research.

Previous studies have consistently shown that elevated blood Glu and lipid levels are positively correlated with increased GDF15 concentrations (17), which aligns with our findings. Recent studies further suggest that GDF15 is closely associated with metabolic conditions such as diabetes and hyperlipidemia, in which higher circulating GDF15 levels have been observed (24). In hyperglycemic conditions, cellular stress responses lead to endothelial dysfunction and a heightened risk of microthrombus formation (25). As a validated clinical indicator of insulin resistance, the TyG index is significantly negatively correlated with circulating GDF15 levels. These results are consistent with previous studies indicating GDF15 improves insulin sensitivity through GRFAL receptor interactions and *β*-adrenergic signaling pathways during metabolic stress (26). In addition, TG, Glu, and GDF15 levels were independently associated with RAO. Notably, our previous study demonstrated that HDL-ch and apolipoprotein A1 (ApoA1) possess strong predictive value for RAO (27, 28). Additionally, we observed a negative correlation between eGFR and GDF15, with the decline in eGFR likely resulting from renal microvascular injury secondary to hyperglycemia-induced microangiopathy and inflammation (29, 30). Increased GDF15 levels have been observed in cases of diabetic nephropathy (31), where GDF15 serves as both a biomarker of disease progression and a protective mediator by modulating inflammatory pathways (32, 33). Collectively, these findings suggest that increased GDF15 levels in RAO patients may reflect underlying renal microvascular dysfunction, corroborating prior evidence that diabetic nephropathy commonly coexists with RAO (34, 35).

Extensive previous studies have demonstrated close epidemiological and pathological associations between RAO and systemic diseases such as diabetes mellitus (36) and cardiovascular diseases (13). RAO is not merely an acute ophthalmic emergency but is increasingly recognized as a potential early indicator of severe cardiovascular and cerebrovascular events, including myocardial infarction and stroke (18, 37). In 2021, the American Heart Association (AHA) scientific statement highlighted that CRAO patients with concomitant atherosclerosis and hypertension carry a poor prognosis, as CRAO is associated with a significantly increased short-term risk of ischemic stroke (approximately 1–2% within 7 days and ~2–3% within 30 days) (13). As a well-established stress-responsive cytokine, GDF15 serves as a biomarker for a spectrum of metabolic and cardiovascular diseases, including diabetes (38), hypertension (39), and stroke (18), with evidence pointing to its causal role in diabetes (40). As a key marker of stress and inflammatory responses, GDF15 may reflect a systemic pathological state characterized by heightened stress responses and vascular dysfunction (41). From this perspective, elevated GDF15 levels in RAO may not be limited to local retinal ischemic injury but instead indicate a broader systemic condition associated with increased cardiovascular risk. However, in the present study, we did not observe a significant association between GDF15 levels and the occurrence of systemic complications. This negative finding may be attributable to the limited sample size or the inherent constraints of the cross-sectional study design. Future studies with larger sample sizes, multicenter cohorts, and prospective follow-up are warranted to further elucidate the role of GDF15 levels in assessing the systemic complication risk in RAO.

Our study is the first to investigate the association between serum GDF15 levels and the prevalence of RAO, offering compelling evidence for its potential as a diagnostic biomarker. By utilizing a matched case–control design, we enhanced internal validity and minimized confounding factors, thereby strengthening the robustness of our findings. However, several limitations warrant consideration. As a cross-sectional study, our analysis cannot establish causality, and the role of GDF15 levels in the pathophysiological mechanisms of RAO remains unclear. The single-center design, based on patients from a single institution in Hubei Province, thus the applicability of these results could be restricted. Despite including nearly all available cases, the rarity of RAO resulted in a sample size insufficient for detailed subgroup analyses, such as stratification by RAO subtype. Although propensity score matching improved group comparability, the high prevalence of comorbid conditions, particularly diabetes-related hypertension, may have influenced outcomes. Subsequent multicenter studies with prospective designs are needed to clarify whether GDF15 levels can serve as a biomarker for identifying RAO-related systemic complications. In addition, mechanistic insights into the role of GDF15 levels in RAO are limited in the present study. Further investigations using animal and cellular models are needed to clarify the underlying mechanisms.

## 5 Conclusion

This study showed that serum GDF15 levels were significantly higher in patients with RAO and were independently associated with RAO risk in multivariate analyses, supporting its potential utility as a biomarker. Combining GDF15 with routine clinical indicators may facilitate RAO identification.

## Data Availability

The raw data supporting the conclusions of this article will be made available by the authors, without undue reservation.
