# Supplementary material for: Association of elevated circulating GDF15 and risk in acute retinal artery occlusion
Source: Front Neurol. 2026 Mar 16;17:1707237. doi: 10.3389/fneur.2026.1707237 (PMC13033525; doi:10.3389/fneur.2026.1707237)
Supplement: Supplementary file 1 [file Table_1.docx]

**Supplementary table 1. Subgroup analyses of circulating GDF15 levels according to hypertension and diabetes status**

| Subgroup | RAO (n) | Control (n) | RAO (mean ± SD, pg/mL) | Control (mean ± SD, pg/mL) | Difference in means (pg/mL) | 95% CI (pg/mL) | P value |
| --- | --- | --- | --- | --- | --- | --- | --- |
| Hypertension |  |  |  |  |  |  |  |
| No | 44 | 82 | 600.880 ± 302.380 | 382.080 ± 221.740 | 218.80 | 125.350–312.240 | <0.001 |
| Yes | 61 | 21 | 689.930 ± 394.930 | 420.630 ± 169.960 | 269.30 | 91.860–446.740 | 0.003 |
| Diabetes |  |  |  |  |  |  |  |
| No | 90 | 99 | 591.250 ± 290.720 | 375.060 ± 171.490 | 216.19 | 148.420–283.960 | <0.001 |
| Yes | 15 | 5 | 1020.750 ± 506.990 | 728.370 ± 537.780 | 292.38 | −850.020–265.250 | 0.290 |

Supplementary table 2. Baseline characteristics of CRAO and BRAO subgroups

| Characteristics | CRAO  (n =93) | BRAO  (n =12) | P value |
| --- | --- | --- | --- |
| Clinical variables |  |  |  |
| Male sex (%) | 72.00 | 66.70 | 0.698 |
| Age (years) | 58.00(50.00，67.00） | 63.50（54.00，66.00） | 0.206 |
| Hypertension (%) | 54.80 | 83.30 | 0.060 |
| Diabetes (%) | 12.90 | 25.00 | 0.260 |
| Laboratory variables |  |  |  |
| WBC (x10^9^/L) | 6.53(5.41 ，8.07） | 5.77（4.70，6.42） | 0.113 |
| Neu (x10^9^/L) | 3.73(2.97 ，4.79） | 3.41（2.92，4.23） | 0.351 |
| Lym (x10^9^/L) | 1.84(1.51 ，2.36） | 1.425（1.14，1.98） | 0.029 |
| Mono (x10^9^/L) | 0.50(0.40 ，0.59） | 0.51（0.38，0.64） | 0.868 |
| ALT (U/L) | 18.00(13.00，26.00） | 21.50（10.00，38.75） | 0.680 |
| AST (U/L) | 18.50(16.00，23.75） | 18.50（14.00，30.50） | 0.681 |
| ALT/AST | 1.00(0.76，1.24） | 1.00（0.76，1.39） | 0.436 |
| Tch (mmol/L) | 4.57(4.00，5.28） | 4.25（3.49，5.10） | 0.991 |
| TG (mmol/L) | 1.57(1.03，2.03） | 1.60（1.04，3.59） | 0.306 |
| HDL-ch (mmol/L) | 1.02(0.86，1.16） | 0.86（0.78，1.10） | 0.945 |
| LDL-ch (mmol/L) | 2.83(2.33，3.36） | 2.34（1.59，3.08） | 0.660 |
| Tch/HDL-ch | 4.52(3.77，5.31） | 5.01（3.45，5.60） | 0.165 |
| Urea (mmol/L) | 5.57(4.80，6.47） | 5.85（4.63，7.06） | 0.334 |
| Cr(μmol/L) | 69.50(58.00，80.75） | 73.00（62.75，88.50） | 0.165 |
| Urea/Cr | 11.89(9.66，14.89） | 11.11（9.46，15.52） | 0.060 |
| Glu (mmol/L) | 5.31(4.92 ，5.97） | 5.02（4.61，5.88） | 0.789 |
| eGFR (ml/min/1.73m^2^) | 95.73(86.73， 105.14） | 91.37（77.08，99.31） | 0.210 |
| TYG index | 8.89(8.39 ，9.19） | 8.72（8.42，9.75） | 0.545 |
| GDF15 (pg/ml) | 587.90(378.67 ，775.62） | 642.82（425.99，1168.58） | 0.242 |

Data are expressed as percent (n) or median (interquartile range).

**CRAO:** central retinal artery occlusion; **BRAO:** branch retinal artery occlusion; **WBC**: white blood cell; **Neu**: neutrophil; **Lym**: lymphocyte; **Mono**: monocyte; **ALT**: glutamic pyruvic transaminase; **AST**: glutamic oxaloacetic transaminase; **ALT/AST**: glutamic pyruvic transaminase-to -glutamic oxaloacetic transaminase ratio; **Tch**: total cholesterol; TG: triglycerides; **HDL-ch**: high-density lipoprotein cholesterol; **LDL-ch**: low-density lipoprotein cholesterol; **eGFR**: estimated glomerular filtration rate; **Glu**: glucose. **TYG** (Triglyceride-Glucose Index) is calculated using the formula: TYG Index = ln[(Fasting Triglycerides (mg/dL) × Fasting Glucose (mg/dL) / 2]; **GDF15**: growth differentiation factor 15

**Supplementary table 3** Collinearity diagnostics of variables included in the multivariable model

| Characteristics | Tolerance | VIF |
| --- | --- | --- |
| Hypertension (%) | 0.832 | 1.202 |
| Diabetes (%) | 0.734 | 1.362 |
| WBC (x10^9^/L) | 0.927 | 1.079 |
| TG (mmol/L) | 0.848 | 1.179 |
| Glu (mmol/L) | 0.746 | 1.341 |
| Urea (mmol/L) | 0.859 | 1.164 |
| eGFR (ml/min/1.73m^2^) | 0.933 | 1.072 |
| GDF15 (pg/ml) | 0.676 | 1.479 |

**VIF：**Variance Inflation Factor；**WBC**: white blood cell; **Neu**: neutrophil; **Lym**: lymphocyte; **Mono**: monocyte; **ALT**: glutamic pyruvic transaminase; **AST**: glutamic oxaloacetic transaminase; **Tch**: total cholesterol; **TG**: triglycerides; **Glu**: glucose. **eGFR**: estimated glomerular filtration rate; **TYG** (Triglyceride-Glucose Index) is calculated using the formula: TYG Index = ln[(Fasting Triglycerides (mg/dL) × Fasting Glucose (mg/dL) / 2]; **GDF15**: growth differentiation factor 15
